# DICER1-синдром с дебютом эмбриональной рабдомиосаркомы гениталий на первом году жизни: описание клинического случая

**DOI:** 10.14341/probl13501

**Published:** 2025-07-22

**Authors:** И. Г. Сичинава, Е. С. Демина, Е. М. Шарибжанова, Ф. К. Исмаилова, А. Г. Гвоздкова, Д. О. Коростин, Е. Е. Петряйкина, А. Н. Тюльпаков

**Affiliations:** Российский национальный исследовательский медицинский университет имени Н.И. Пирогова; Российский национальный исследовательский медицинский университет имени Н.И. Пирогова; Российская детская клиническая больница; Российская детская клиническая больница; Российский национальный исследовательский медицинский университет имени Н.И. Пирогова; Российский национальный исследовательский медицинский университет имени Н.И. Пирогова; Центр высокоточного редактирования и генетических технологий для биомедицины; Российский национальный исследовательский медицинский университет имени Н.И. Пирогова; Российская детская клиническая больница; Российская детская клиническая больница; Медико-генетический научный центр им. акад. Н.П. Бочкова

**Keywords:** DICER1-синдром, эмбриональная рабдомиосаркома матки и влагалища, фолликулярная узловая болезнь щитовидной железы

## Abstract

DICER1-синдром — редкое моногенное заболевание с аутосомно-доминантным типом наследования. Белок DICER1 участвует в регуляции экспрессии генов при помощи микроРНК, и нарушение его функции может приводить к возникновению опухолей различной локализации. Представлен клинический случай эмбриональной рабдомиосаркомы (ЭРМС) матки и влагалища, дебютировавшей у пациентки в возрасте 6 мес с последующим развитием фолликулярной узловой болезни щитовидной железы (ФУБЩЖ) в 13 лет 9 мес. При проведении молекулярно-генетического обследования выявлен гетерозиготный патогенный вариант p.Arg1003Ter в гене DICER1 (NM_030621.4). Настоящее наблюдение подчеркивает важность соблюдения четкого диагностического алгоритма при ведении пациентов с ФУБЩЖ и злокачественными новообразованиями в анамнезе, а именно проведения своевременной молекулярно-генетической диагностики DICER1-синдрома. ЭРМС гениталий следует рассматривать как вероятный компонент DICER1-синдрома, после подтверждения которого необходим скрининг других проявлений синдрома (заболевания).

## Abstract (en)

DICER1 syndrome is a rare monogenic disease with autosomal dominant inheritance. DICER1 protein is involved in the regulation of gene expression by microRNAs. Changes in the expression of DICER1 can be associated with various cancers. A 13,8-year-old girl with a history of embryonal rhabdomyosarcoma (ERMS) of uterine cervix and vagina excised at 6 months of age is presented with a thyroid follicular nodular disease (TFND). Molecular genetic examination revealed a heterozygous pathogenic variant p.Arg1003Ter in the DICER1 gene (NM_030621.4). The presented case emphasizes the importance of molecular genetic diagnosis of DICER1 syndrome in a diagnostic algorithm in the management of patients with TFND and history of malignancy. Considering ERMS of genital tract as a probable component of DICER1 syndrome it is necessary to screen for other manifestations of the disease as well.

## АКТУАЛЬНОСТЬ

Онкопатология занимает одно из ведущих мест в структуре заболеваемости и смертности во всем мире, что делает чрезвычайно актуальным изучение молекулярных механизмов развития опухолей, открывающее перспективы для совершенствования ранней диагностики и создания новых подходов к лечению. Особое место в структуре онкологических болезней занимают наследственные синдромы, компонентом которых нередко являются эндокринные опухоли. К числу таких состояний относится DICER1-синдром — редкое моногенное заболевание с аутосомно-доминантным типом наследования, которое обусловлено мутациями в одноименном гене. Белок DICER1 участвует в регуляции экспрессии генов при помощи микроРНК, нарушение его функции может приводить к неконтролируемой пролиферации и возникновению опухолей различной локализации. Самым частым проявлением DICER1-синдрома является фолликулярная узловая болезнь щитовидной железы (ФУБЩЖ), которая может быть единственным проявлением синдрома, а также сочетаться с другими его компонентами. В настоящей публикации нами представлено описание случая ФУБЩЖ у 13-летней пациентки, у которой DICER1-синдром манифестировал в возрасте 6 мес эмбриональной рабдомиосаркомой (ЭРМС) матки и влагалища.

## ОПИСАНИЕ СЛУЧАЯ

Пациентка Р. поступила в эндокринологическое отделение РДКБ в возрасте 14 лет 9 мес с направляющим диагнозом: «Нетоксический многоузловой зоб».

Из анамнеза известно, что ребенок от третьей беременности, протекавшей на фоне артериальной гипертензии, третьих своевременных оперативных родов (КС). Вес ребенка при рождении — 3200 г, длина — 50 см. Оценка по шкале Апгар 7/8 баллов. На первом году жизни наблюдалась неврологом с диагнозом: «Перинатальное поражение ЦНC».

Наследственный анамнез отягощен: заболевание щитовидной железы у матери — точные данные отсутствуют, принимает левотироксин натрия; у тети по материнской линии тиреоидэктомия по поводу доброкачественного узла; брат и сестра, со слов матери, здоровы.

В возрасте 6 мес впервые мамой обнаружено опухолевидное образование в области наружных половых органов, которое было удалено; по данным гистологии — капиллярная гемангиома. В возрасте 8 мес в связи с задержкой мочеиспускания девочка была госпитализирована в хирургическое отделение, где в области половой щели обнаружено опухолевидное образование, сдавливающее окружающие ткани. Часть опухоли удалена, по результатам гистологического исследования — ботриоидная рабдомиосаркома. После 4 курсов ПХТ в возрасте 11 мес была проведена лапаротомия, экстирпация матки и влагалища с опухолью. В гистопрепаратах тела матки и стенок влагалища определяется опухолевая ткань, имеющая строение ботриоидной рабдомиосаркомы с явлениями терапевтического патоморфоза 2 степени. В гистопрепаратах яичника опухолевых клеток не выявлено. В послеоперационном периоде дополнительно проведено 5 курсов ПХТ и курс лучевой терапии.

В 13 лет 9 мес впервые при УЗИ щитовидной железы были выявлены множественные узлы (единичное узловое образование в правой доле 12,0х6,0х8,0 мм, а также два образования в левой доле 10,0х7,5х11,0 мм и 12,0х8,5х9,5 мм) общий объем железы — 7,7 см³, TIRADS-III. Региональные лимфатические узлы не изменены. В гормональном профиле: ТТГ — 1,3 мЕд/л (норма 0,4–4,0); свТ4 — 11,0 пмоль/л (норма 10,48–17,29); свТ3 — 5,4 пмоль/л (3,34-5,79); кальцитонин <2 пг/мл (норма <11,5); тиреоглобулин — 23,39 нг/мл (норма 1,59–50,0). При исследовании онкомаркеров патологии выявлено не было.

При поступлении: состояние удовлетворительное. SDS роста=-3,49; SDS ИМТ=+2,1. Послеоперационный рубец на передней брюшной стенке — без признаков келоида. Нарушение осанки по сколиотическому типу. По внутренним органам — без особенностей. Щитовидная железа пальпаторно — 1 степень по ВОЗ, клинически: эутиреоз. Наружные половые органы сформированы по женскому типу. Таннер 4. Менструации отсутствуют.

При УЗИ щитовидной железы размеры не увеличены, объем — 7,7 см³. Перешеек — 2 мм. Правая доля 40х12х19 мм (4,4 см³), левая доля 36х12х16 мм (3,3 см³). Контуры неровные, четкие. Эхогенность паренхимы не изменена, структура однородная. Васкуляризация при ЦДК не изменена. В центре и в верхнем полюсе по задней поверхности правой доли визуализируется эхонегативное кистозное образование 24х8х15 мм и 5х4х8 мм без эффекта усиления У3-сигнала, с однородным содержимым и ровными четкими контурами, множество мелких кист. В левой доле кисты размерами 10х7х9 мм и 8,5х4,5х7 мм, множество мелких кист. В проекции паращитовидных желез патологических эхо-сигналов не выявлено. Регионарные лимфатические узлы не изменены. Заключение: множественные кистозные образования (коллоидные) обеих долей щитовидной железы, TIRADS II.

УЗИ органов малого таза: матка и влагалище удалены; правый яичник 21х10х18 мм с фолликулами до 3 мм, левый яичник не визуализируется. Свободная жидкость и дополнительные образования в малом тазу не выявлены, выраженный спаечный процесс. Регионарные лимфатические узлы не изменены. Заключение: состояние после экстирпации матки и влагалища.

Гормональный профиль: ЛГ — 2,08 МЕ/л (норма 0,5–13,1); ФСГ — 6,36 МЕ/л (норма 0,3–7,8); тестостерон — 0,64 нмоль/л (норма 0,49–1,7); эстрадиол — 164,16 пмоль/л (норма 0–936); ТТГ — 1,29 мЕд/л (норма 0,4–4,0); свТ4 — 11,48 пмоль/л (10,48–17,29); свТ3 — 4,63 пмоль/л (3,34–5,79); АТ-ТПО — 0,39 МЕ/мл (0–5,61); кальцитонин — 1,10 пг/мл (норма 0,50–6,40); тиреоглобулин — 23,71 нг/мл (норма 3,50–77,0).

Для уточнения диагноза проведена молекулярно-генетическая диагностика. Полноэкзомное секвенирование осуществлялось на приборе G-400 (MGI Tech) с использованием ранее описанного лабораторного протокола [1]. Интерпретация клинической значимости выявленных вариантов производилась в соответствии с критериями ACMG [2][3] с использованием баз данных вариантов и литературных источников.

В результате ДНК-анализа у пациентки обнаружен вариант нуклеотидной последовательности в экзоне 21 гена DICER1 (chr14:95572101G>A) в гетерозиготном состоянии, приводящий к появлению сайта преждевременной терминации трансляции в кодоне 1003 (p.Arg1003Ter, NM_030621.4). При обследовании матери данный вариант обнаружен не был.

Выявленный нами вариант p.Arg1003Ter был ранее описан у пациентки, у которой было диагностировано 3 компонента DICER1-синдрома: ФУБЩЖ и плевролегочная бластома (в возрасте 16 лет) и эндометриальная аденосаркома (в 27 лет) [4].

## ОБСУЖДЕНИЕ

Ген DICER1 ген локализован на длинном плече 14 хромосомы (14q32.13) и кодирует 1922-аминокислотный белок DICER1, также известный как рибонуклеаза класса 3 (RNase III) [5]. В состав белка входят несколько функциональных доменов, в том числе: хеликазные домены DExD/Hbox и HELICc; домен TRBP-BD, отвечающий за связывание РНК-связывающего белка в ответ на трансактивацию; рибонуклеазные домены RNase IIIa и RNase IIIb; и домен dsRBD, отвечающий за связывание двухцепочечной РНК [6].

Белок играет одну из ключевых ролей в так называемой РНК-интерференции — процессе подавления экспрессии гена при помощи малых молекул РНК. DICER1 отвечает за процессинг кодируемых таргетными генами молекул предшественников микроРНК (премикроРНК) в зрелые микроРНК, превращении двухцепочечных РНК в малые интерферирующие РНК, а также участвует в сборке и функционировании РНК-белковых комплексов RISC [6].

Впервые пациенты с DICER1-синдромом были описаны O’Brien и Wilansky [7], которые заподозрили наследственный механизм развития опухолей при сочетании многоузлового зоба (в соответствии с новой классификацией [8], фолликулярная узловая болезнь щитовидной железы, ФУБЩЖ) и опухолей из клеток Сертоли-Лейдига. Между тем герминальные мутации в гене DICER1 как доказанная причина наследственной опухоли были впервые выявлены при другой патологии — плевролегочной бластоме (ПЛБ) [9]. Hill с соавт. при обследовании семей, для которых ранее было описано сочетание ПЛБ с другими опухолями [10], используя метод позиционного клонирования, установили связь патологии с локусом гена DICER1 и выявили гетерозиготные герминальные мутации в данном гене во всех семьях, включенных в исследование [9]. ПЛБ относится к частым проявлениям DICER1-синдрома, как и опухоли яичников из клеток Сертоли-Лейдига, ФУБЩЖ и кистозная нефрома [6][11]. К более редким компонентам синдрома относятся эмбриональная рабдомиосаркома (ЭРМС) с различной локализацией, дифференцированная тиреокарцинома, опухоль Вильмса, ювенильный гамартоматозный полипоз кишечника, медуллоэпителиома циалиарного тела, назальная хондромезинхимальная гамартома, бластома гипофиза и пинеобластома [6]. Распространенность DICER1-синдрома точно неизвестна, однако показано, что популяционная частота патогенных вариантов в гене DICER1 составляет 1:10600 [12]. Как и при других наследственных опухолевых синдромах, риск развития опухолей при DICER1-синдроме увеличивается с возрастом и составляет в 10 и 60 лет 5,3 и 31,5% в сравнении с общепопуляционным риском для данных возрастных групп 0,17% и 6,6% соответственно [13][14].

ЭРМС как компонент DICER1-синдрома впервые был упомянут Foulkes с соавт. при описании 7 семей, в 3 из которых было отмечено сочетание ЭРМС шейки матки и ФУБЩЖ, диагностированных в возрасте 14–17 лет и 14–19 лет соответственно [15]. Возраст дебюта ЭРМС различной локализации значительно варьирует. Показано, что ботриоидная ЭРМС с влагалищной локализацией выявляется преимущественно у детей раннего возраста (0–9 лет, медиана — 21 мес), тогда как опухоли с цервикальной локализацией характерны для более старшей возрастной группы (11–19 лет, медиана — 15 лет) [16][17]. Следует также отметить, что вагинальные ЭРМС крайне редко ассоциируют с DICER1-синдромом. Так, в обзоре Apellaniz-Ruiz с соавт. приводятся данные по 46 случаям ЭРМС генитального тракта, ассоциированным с DICER1-синдромом, среди которых было только 3 пациента с опухолью влагалища в возрасте 5, 13 и 14 лет [18]. В доступных научно-медицинских источниках мы не нашли описания DICER1-ассоциированных ЭРМС влагалища у детей первого года жизни. Именно ботриоидная ЭРМС была выявлена у нашей пациентки в возрасте 6 мес. Данная форма заболевания, как правило, сопровождается вагинальным кровотечением, болью и опухолевым разрастанием, что и было причиной первого экстренного обращения родителей пациентки к врачам.

ФУБЩЖ является самым частым проявлением DICER1-синдрома [11], которое обычно манифестирует в возрасте от 10 до 30 лет и чаще наблюдается у женщин [6][11]. Сама по себе ФУБЩЖ не является специфичной для DICER1-синдрома, но выявление ее в детском возрасте и в сочетании с любым другим компонентом синдрома с большой вероятностью указывает на связь с DICER1 [19]. К более редким вариантам тиреоидной патологии, ассоциированной с DICER1-синдромом, относятся фолликулярная аденома с папиллярной архитектурой, фолликулярная карцинома щитовидной железы и папиллярная карцинома щитовидной железы [20][21]. Несмотря на то, что при DICER1-синдроме риск развития тиреокарциномы увеличивается в 16–24 раза [22], большинство экспертов считают, что тактика наблюдения пациентов не должна отличаться от таковой при других узловых образованиях щитовидной железы, и сам факт выявления герминальной мутации в гене DICER1 не является показанием для тиреоидэктомии [22][23]. У пациентов с ФУБЩЖ предлагается проводить УЗИ щитовидной железы 1 раз в 6 мес, по результатам которого определяются показания к ТАБ и дальнейшим вмешательствам [22][23]. Рекомендуемая тактика также была выбрана для наблюдения за пациенткой, описанной в данной статье.

## ЗАКЛЮЧЕНИЕ

Нами представлен клинический случай, описывающий пациентку с двумя опухолевыми заболеваниями: ЭРМС матки и влагалища и ФУБЩЖ, манифестировавшие в возрасте 6 мес и 13 лет 9 мес соответственно. Сочетание указанных опухолей с выявленной у пациентки герминальной мутацией в гене DICER1 с высокой вероятностью позволяет расценивать их как проявление DICER1-синдрома. Представленное наблюдение свидетельствует о том, что ЭРМС гениталий следует рассматривать как вероятный компонент DICER1-синдрома, что требует проведения соответствующей молекулярной диагностики и, в случае подтверждения, скрининга других проявлений синдрома, в том числе эндокринных опухолей.

## ДОПОЛНИТЕЛЬНАЯ ИНФОРМАЦИЯ

Источники финансирования. Работа выполнена по инициативе авторов без привлечения финансирования.

Конфликт интересов. Авторы декларируют отсутствие явных и потенциальных конфликтов интересов, связанных с содержанием настоящей статьи.

Участие авторов. Сичинава И.Г. — анализ данных, интерпретация результатов, подготовка финальной версии статьи; Дёмина Е.С., Шарибжанова Е.М. — получение и анализ данных, редактирование текста; Исмаилова Ф.К., Гвоздкова А.Г. — сбор и подготовка данных, написание первичного варианта статьи; Коростин Д.О. — проведение молекулярно-генетического исследования; Петряйкина Е.Е. — редактирование текста, внесение ценных замечаний; Тюльпаков А.Н. — концепция и дизайн исследования, интерпретация результатов, внесение существенной правки с целью повышения научной ценности статьи.

Все авторы одобрили финальную версию статьи перед публикацией, выразили согласие нести ответственность за все аспекты работы, подразумевающую надлежащее изучение и решение вопросов, связанных с точностью или добросовестностью любой части работы.

Согласие пациента. Законный представитель пациента добровольно подписал информированное согласие на публикацию персональной медицинской информации в обезличенной форме в журнале «Проблемы эндокринологии».
